# Four Weeks of Balance Training does not Affect Ankle Joint Stiffness in Subjects with Unilateral Chronic Ankle Instability

**DOI:** 10.23937/2469-5718/1510036

**Published:** 2016-01-15

**Authors:** Tarang Kumar Jain, Clayton N. Wauneka, Wen Liu

**Affiliations:** 1 Department of Physical Therapy and Athletic Training, Northern Arizona University, USA; 2 Bioengineering Graduate Program, University of Kansas, Lawrence, USA; 3 Department of Physical Therapy and Rehabilitation Science, University of Kansas Medical Center, USA

**Keywords:** Ankle sprains, Ankle instability, Ankle joint laxity, Rehabilitation

## Abstract

**Background:**

Balance training has been shown to be effective in preventing ankle sprain recurrences in subjects with chronic ankle instability (CAI) but the biomechanical pathways underlying the clinical outcomes are still unknown. This study was conducted to determine if a 4-week balance training intervention can alter the mechanical characteristics in ankles with CAI.

**Methods:**

Twenty-two recreationally active subjects with unilateral CAI were randomized to either a control (n = 11, 35.1 ± 9.3 years) or intervention (n = 11, 33.5 ± 6.6 years) group. Subjects in the intervention group were trained on the affected limb with static and dynamic components using a Biodex balance stability system for 4-weeks. The ankle joint stiffness and neutral zone in inversion and eversion directions on the involved and uninvolved limbs was measured at baseline and post-intervention using a dynamometer.

**Results:**

At baseline, the mean values of the inversion stiffness (0.69 ± 0.37 Nm/degree) in the involved ankle was significantly lower (*p* < 0.011, 95% CI [0.563, 0.544]) than that of uninvolved contralateral ankle (0.99 ± 0.41 Nm/degree). With the available sample size, the eversion stiffness, inversion neutral zone, and eversion neutral zone were not found to be significantly different between the involved and uninvolved contralateral ankles. The 4-week balance training intervention failed to show any significant effect on the passive ankle stiffness and neutral zones in inversion and eversion.

**Conclusion:**

Decreased inversion stiffness in the involved chronic unstable ankle was found that of uninvolved contralateral ankle. The 4-week balance training program intervention was ineffective in altering the mechanical characteristics of ankles with CAI.

**Level of evidence:**

Randomized controlled clinical trial; Level of evidence, 1.

## Introduction

Lateral ankle sprain is one of the most frequent sports-related injuries, accounting for up to 60% of all athletic injuries [1]. The development of repetitive ankle sprains and persistent residual symptoms such as repeated episodes of ankle giving way, pain, weakness, loss of function, and feeling of ankle instability after injury has been termed chronic ankle instability (CAI) [2]. CAI can be caused by either mechanical ankle instability (MAI), functional ankle instability (FAI), or both. Mechanical instability has been defined as “ankle movement beyond the physiologic limit of the ankle’s range of motion” [2] and is frequently quantified through the measurement of joint flexibility. During an ankle sprain, ligaments supporting the ankle joint are stretched beyond their physiological limits, resulting in damage to the fibrous integrity of the ligaments including the anterior talofibular ligament (ATFL), posterior talofibular ligament (PTFL), and/or calaneofibular ligament (CFL) [3]. The damage and incomplete healing of the lateral ligaments of the ankle can lead to increased amount of accessory movement at the joint causing an enlargement of the neutral zone and an abnormal pattern of joint movement [4,5]. The neutral zone is defined as the area of the joint where accessory movement is available without ligamentous lengthening [4,5]. The neutral zone is that part of the range of physiological ankle joint motion, measured from the neutral position, within which the ankle motion is produced with a minimal internal resistance. The signs and symptoms of initial injury often resolve with time but mechanical joint laxity may last longer leading to residual symptoms. Researchers have often relied on the quantity of motion and the amount of resistance at the extreme of passive physiological motion to determine the flexibility characteristics of the ankle joint. Previous *in vivo* studies have indicated that there is higher reliability in assessing the amount of resistance at the extreme of passive physiological motion than assessing range of motion [6]. These results indicate that ligament laxity can be indirectly evaluated through the measurement of the passive joint stiffness (a measure of resistance to stretch). The average load-displacement characteristics (moment relative to angular displacement) can be used to demonstrate the neutral zone and non-linear behavior of the passive resistance with increasing range of motion. A high flexibility around the neutral position and a stiffening effect toward the end of the range of motion contributes to the non-linear load-displacement curve (Figure 3).

Increased mechanical joint laxity has frequently been associated with CAI [7]. Recently, Hubbard et al. [8] also identified mechanical laxity to be the largest predictor in the development of CAI, explaining 31.3% of the variance in individuals with CAI. However, many researchers have also demonstrated that there is no one-to-one association between the ankle joint laxity and CAI [9–13]. Konradsen et al. [12] showed that ankle joint laxity was not associated with a proprioceptive sensory deficit or reduction in muscle strength when compared with the injuries that did not result in ankle laxity. Furthermore, many patients with functional ankle instability did not show any sign of the ankle joint laxity using various diagnostic methods [10,11,13]. The issue of mechanical instability in CAI remains inconclusive due to inconsistent findings in the literature. For instance, Kovaleski et al. [11] measured the maximum passive inversion range of motion and peak passive resistive torque in a group of patients with functional ankle instability and found the two variables to be not significantly different between involved and uninvolved ankles. It is further surprising to see that no study has investigated the neutral zone in patients with CAI to date. Although there is no conclusive evidence, based on spinal instability studies, it can be assumed that an increase in neutral zone can lead to early ankle joint degeneration and repetitive ankle injuries [4]. Excessive ankle laxity is an indication for ligamentous reconstruction or repair and accurate diagnosis of the passive ligamentous laxity in the neutral and elastic zones can prevent an unnecessary surgery, and /or prevent an unnecessary delay in surgery if a surgical intervention is indicated [14].

Balance training has been shown to be effective in preventing ankle sprain recurrences in patients with CAI but the biomechanical pathways underlying the clinical outcomes are still unknown [15]. Balance training is routinely used in clinical practice for sprained ankles, however to our knowledge, only one study has examined the effects of balance training intervention on flexibility characteristics of the ankle joint in patients with CAI [16]. The study reported no change in joint stiffness after balance training, but did not examine the neutral zone. Therefore, the specific aims of the present study were to compare the flexibility/stiffness and neutral zone between the involved ankle with CAI and contralateral uninvolved ankle, and to determine whether the mechanical characteristics in ankles with CAI can be altered through 4-week balance training intervention.

## Methods

### Experimental design and participants

**Figure 1:**
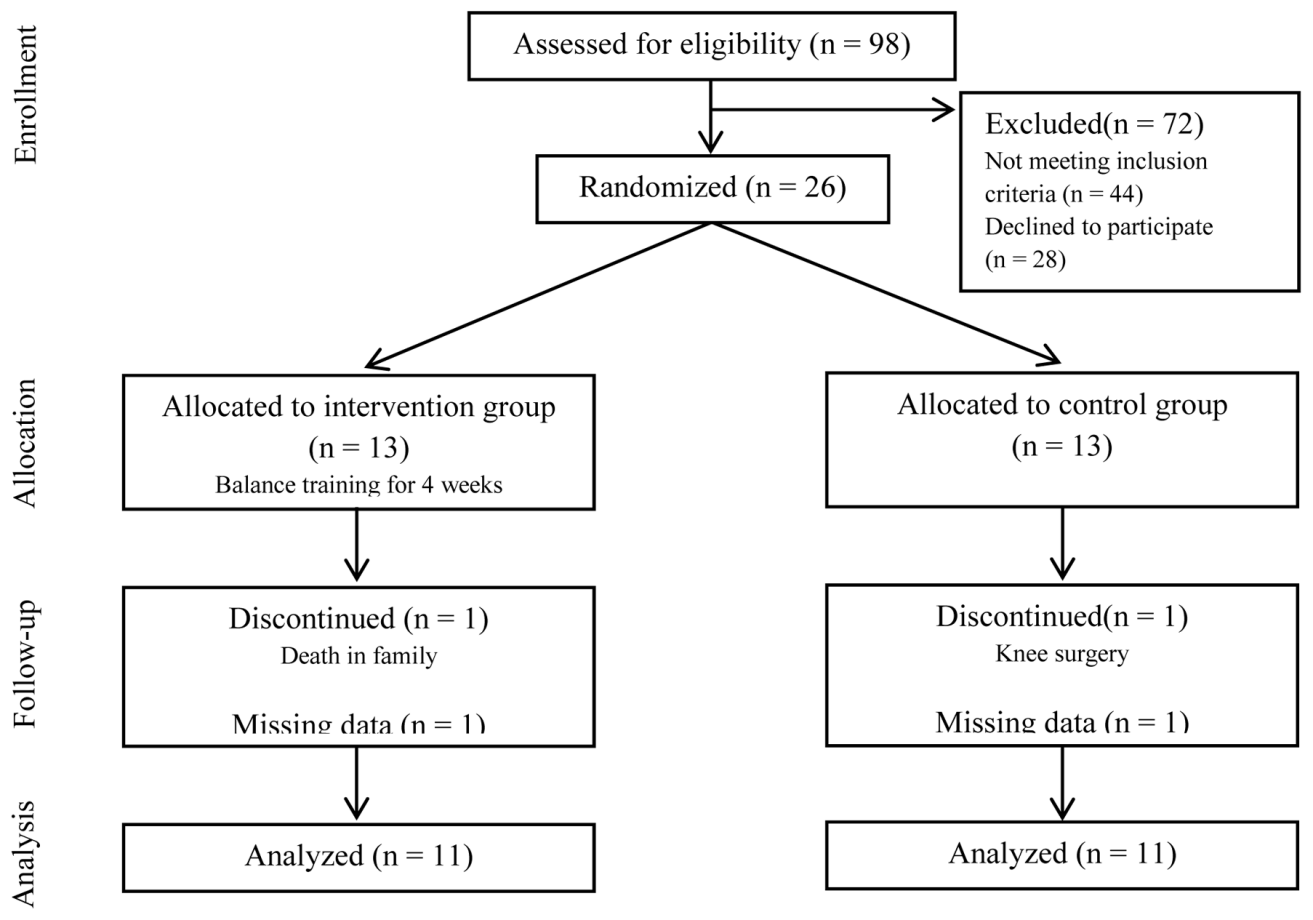
Flow of subjects through the phases of randomized control trial

The present project was a randomized, single-blinded study of balance training program in subjects with CAI. Twenty-six (19 females, 7 males) recreationally active individuals with a history of unilateral CAI (age, 34.2 ± 7.7 years, weight, 75.3 ± 13.6 kg; height, 170 ± 8.8cm) were recruited between March 2010 and August 2013 via flyers, electronic mail, and from the local university employees (Figure 1). In this study, subjects were considered to have chronic ankle instability if they reported ankle giving way episodes and/or recurrent sprains during functional activities for a minimum of 12 months post-initial ankle sprain. All subjects in the study were diagnosed with either a grade 2 or 3 initial lateral ankle sprain by their physician. On further questioning, subjects confirmed that the lateral ankle sprains they experienced were from a plantar-flexion/inversion-type movement.

Before enrolling in the study, potential subjects were screened through the self-reported disability/function questionnaire - Cumberland ankle instability tool (CAIT). Subjects were included in the study if they were between 18 and 45 years of age, had an active range of ankle joint motion of at least 35 degrees of the inversion/eversion and 20 degrees of plantar flexion, presented at least four weeks after an repetitive unilateral ankle inversion sprain (> grade II), self-reported ongoing ankle giving way incidence during functional activities, and active in exercise for at least 2 hours per week. Subjects were excluded if they exhibited any of the following criteria: (1) severe ankle pain and swelling, (2) ankle surgery, (3) gross limitation in ankle motion or inversion range of motion, (4) lower extremity injury other than ankle sprain in past 12 weeks, (5) current enrollment in formal rehabilitation program, (6) history of insulin-dependent diabetes, (7) any systemic disease that might interfere with sensory input or muscle function of the lower extremity, or (8) any joint disease or surgery in the legs. Prior to participation, all subjects signed an informed consent approved by the Institutional Review Board at the University.

Following initial screening, subjects were randomized into 2 groups (intervention and control) using a random allocation sequence list created by a computerized random number generator. Subjects varied in number of ankle sprains, giving-way episodes, self-reported disability/function questionnaire, treatment history, and time since last ankle giving-way episode. The examiners were unaware of the subject group assignment and subjects were also instructed to avoid mentioning details about their study to examiners.

### Instrumentation and procedure

**Figure 2:**
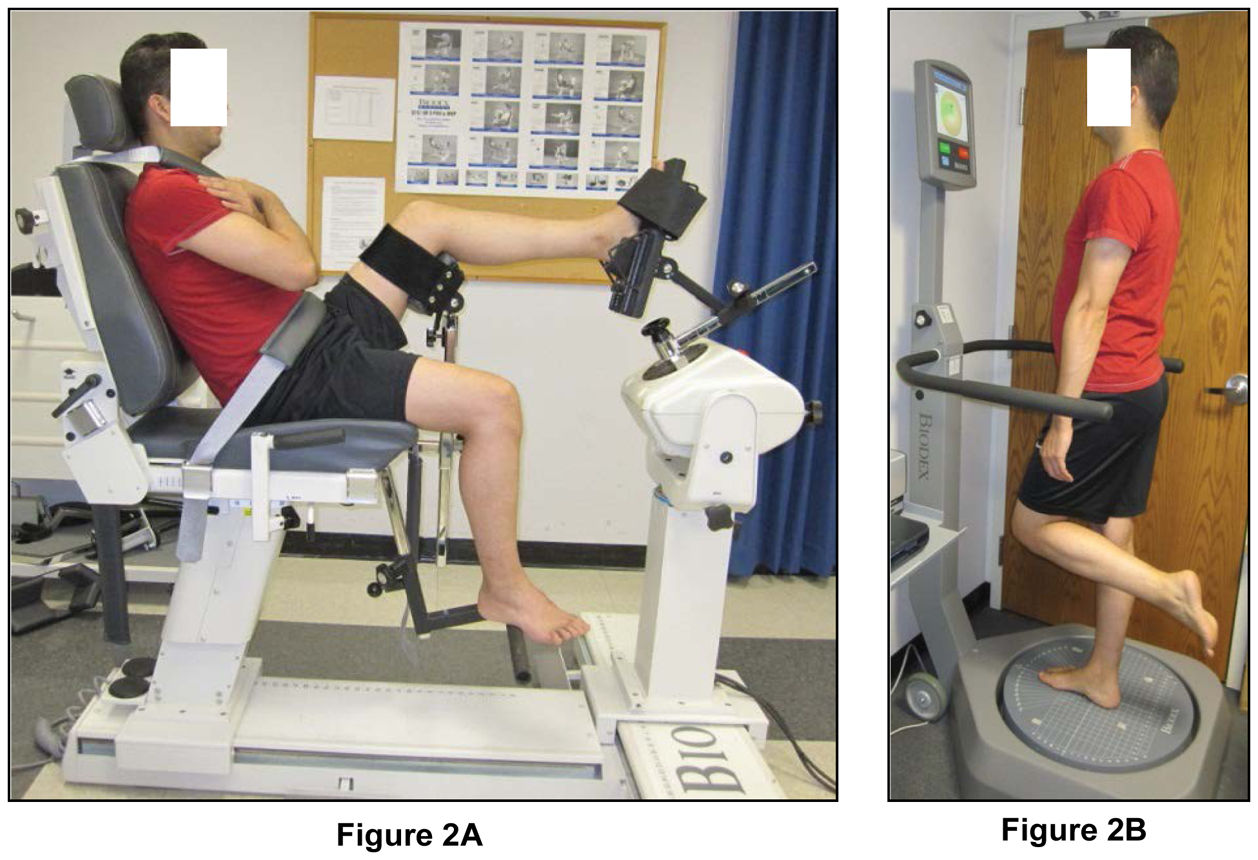
Illustration of stiffness testing and balance training setup. (A) Stiffness testing using Biodex dynamometer; and (B) Balance training using Biodex Balance Stability System.

Ankle joint stiffness and neutral zone in inversion and eversion were assessed using a Biodex System 3 dynamometer (Biodex Medical Systems Inc, Shirley, NY). The dynamometer chair was oriented at 90 degrees and tilted back at 70 degrees. Subject was stabilized and secured with harnesses across the lap and trunk while sitting on the chair. The knee was flexed at approximately 30 degrees and the ankle was set at 20 degrees of plantar flexion. This testing position was chosen because measurement reliability has been shown to be higher in this position compared to neutral [17] and may permit better isolation of the ankle capsule ligamentous structures [18]. Adjustments were made to align the midline of the foot with the midline of the patella, with the entire length of the tibial crest approximating a horizontal orientation. The calf of the tested leg was secured on a 40 cm high platform by hook and loop straps according to the manufacturer’s guidelines. The subject’s talocrural joint axis was aligned with the axis of the dynamometer with the foot held in a posterior heel cup with a strap around the talar head and the forefoot and toes being secured through a dorsal strap (Figure 2A). Similar methods for determination of passive stiffness with high reliability measurements (ICC [2,1] = 0.767 – 0.943) have been reported in the literature [19,20].

All subjects underwent an ankle stiffness test on both ankles in a random order on two different days separated by approximately 3 days. Prior to the passive ankle stiffness testing, a full range of inversion and eversion movement with the ankle at 20 degrees of plantar flexion was determined for each subject based on the subject’s subjective sensation of the maximum attainable movement. The maximum attainable range was determined by asking the subjects to move their ankle voluntarily to maximal eversion to establish the mechanical eversion stop and to inversion to establish the mechanical inversion stop. Once the maximum attainable inversion-eversion range was determined, the dynamometer was calibrated for each subject according to the available range of motion. During the test, the subject’s ankle was positioned in a neutral position of the inversion/eversion and 20 degrees of plantar flexion. The dynamometer then passively rotated the ankle at an angular velocity of 5 degrees per second to the maximum attainable range of motion. The subjects were instructed to relax their ankles and legs and allow their ankle to be moved as far as they can tolerate. The resistive torque during the passive inversion and eversion motion through this maximum attainable range of motion was recorded. The passive motion was repeated for a total of six maximum attainable full range movements.

**Figure 3:**
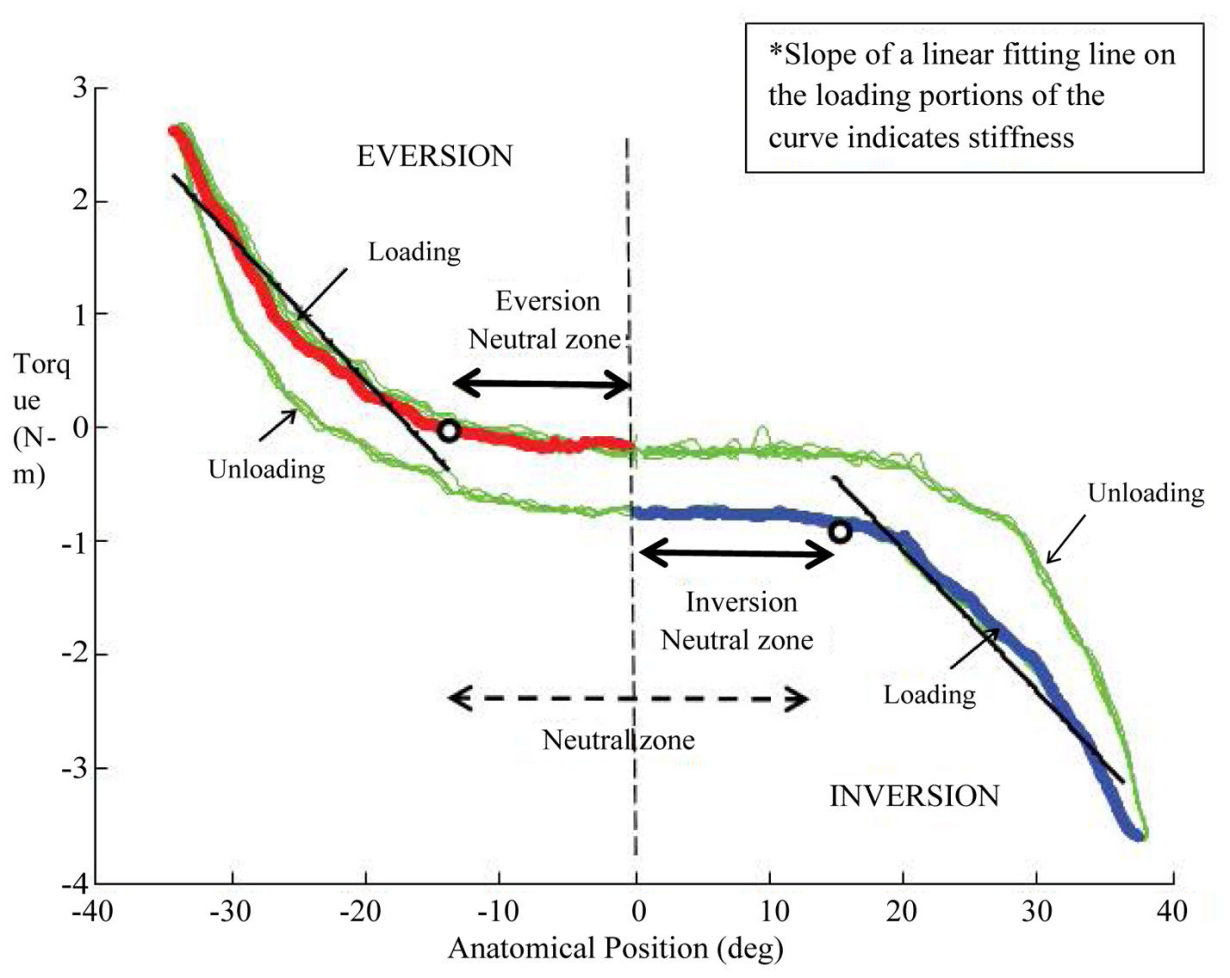
An illustration of stiffness and neutral zone measurement on an angular displacement-moment curve obtained from one subject

The recorded load-displacement curve was processed to obtain two key variables: neutral zone and stiffness of the curve. The neutral zone in inversion and eversion direction was measured as a range between the neutral joint position to the position where a 10% deviation of load occurred in either direction, respectively (Figure 3). The stiffness was measured as the slope of a linear fitting line to loading portion of the load-displacement curve between the end of neutral zone and the maximum of the curve.

Stiffness data was further normalized to calculate the normalized stiffness of the involved ankle in inversion (INS %) and eversion (EVS %) using the formula [(S_involved_ − S_uninvolved_) / S_uninvolved_] × 100, where S is the stiffness in newton-meter/degree. Similarly, neutral zone data was normalized to calculate the normalized neutral zone of the involved ankle in inversion (INNZ %) and eversion (EVNZ %) using the formula [(D_involved_ − D_uninvolved_) / D_uninvolved_] × 100, where D is the neutral zone in degrees. The difference in the normalized values for each dependent variable between the baseline and post-intervention was also calculated to assess the effect of balance training.

### Balance training program

The balance training was performed using a commercially available device, the Biodex Balance Stability System (BSS) (Biodex, Inc., Shirley, New York). The BSS consists of a circular balance platform that provides up to 20° of surface tilt in a 360° range of motion and can move in the anterior–posterior and medial - lateral axes simultaneously (Figure 2B). The BSS also has built in software (Biodex, Version 3.01, Biodex, Inc.) that allows control of the platform’s stability level based on the amount of tilt allowed. The platform stability ranges from level 1 to 12, with level 1 representing the least stable setting and level 12 as the most stable setting. The amount of tilt allowed by the balance platform is determined by the level setting. Visual feedback of the subject’s sway is provided via a monitor mounted on the BSS.

The subjects in the intervention group performed the balance training program for three days per week for 4 weeks, each session lasting approximately 20 minutes. Training included single limb standing in the presence of a physical therapist, similar to a protocol used by Rozzi et al. [21] (Table 1). Subjects were trained on the affected limb using both static and dynamic balance components. During training on both static and dynamic balance components, subjects were instructed to stand barefoot and maintain the same body position at all stability levels.

For the static balance training, subjects performed balance training at both high (stability level 6) and low (stability level 2) resistance-to-platform-tilt levels. The stability levels 6 represented a fairly stable platform surface while level 2 represented an unstable platform surface. During each training session, subjects stood on the involved limb and the unsupported limb was held in a comfortable position so as not to contact the involved limb or the BSS platform. Subjects were instructed to focus on the visual feedback screen in front of them and to maintain the cursor at the center of the screen by adjusting their balance as needed. Subjects performed three 30-second repetitions of static balancing at both stability levels.

During the dynamic balance training the subjects were instructed to actively move the platform and maintain it within a specified range while focusing on the visual feedback screen on the BSS monitor. Subjects were required to actively tilt the platform in both uni-planar (anterior/posterior and medial/lateral) and multi-planar (clockwise and counterclockwise) directions while staying within the boundaries defined by a circular path on the device’s visual feedback screen. Subjects performed 3 sets of 6 repetitions for both anterior/posterior and medial/lateral tilts and 1 set of 10 circle repetitions in both clockwise and counterclockwise circular movements.

## Statistical Analysis

Paired Student *t*-test corrected for α-inflation by the Bonferroni procedure was used to compare each dependent variable (inversion stiffness, eversion stiffness, inversion neutral zone, and eversion neutral zone) between the involved and uninvolved ankle of all subjects at the baseline. Another independent Student *t*-test corrected for α-inflation by the Bonferroni procedure was performed to compare the difference in the normalized values for each dependent variable (INS %, EVS %, INNZ %, and EVNZ %) between the baseline and post-intervention for the independent variable of groups (intervention and control) to assess the effect of balance training. A value of *p* < 0.025 was used as the threshold for statistical significance for all outcome measures (SPSS v20; IBM Inc, Armonk, NY).

## Results

Ninety-eight potential subjects with chronic inversion ankle sprain were screened for eligibility. Twenty-six subjects satisfied all eligibility criteria, signed an informed consent to participate, and were randomized to either the control or intervention group. A flow diagram of subject recruitment and retention is provided in Figure 1. Two subjects did not complete the study, with one in the control group and the other in intervention group. We also lost data from two subjects due to technical errors during subject testing. Final analysis was performed on 11 control subjects (age, 35.1 ± 9.3 years, 37% male, weight, 76.0 ± 14.6 kg; height, 168.4 ± 10.7 cm) and 11 intervention subjects (age, 33.5 ± 6.6 years, 57% male, weight, 77.1 ± 13.2 kg; height, 172.7 ± 6.1 cm) (Table 2). Baseline demographics between groups were not statistically different (p > 0.05). No adverse events were reported during the study period.

**Figure 4:**
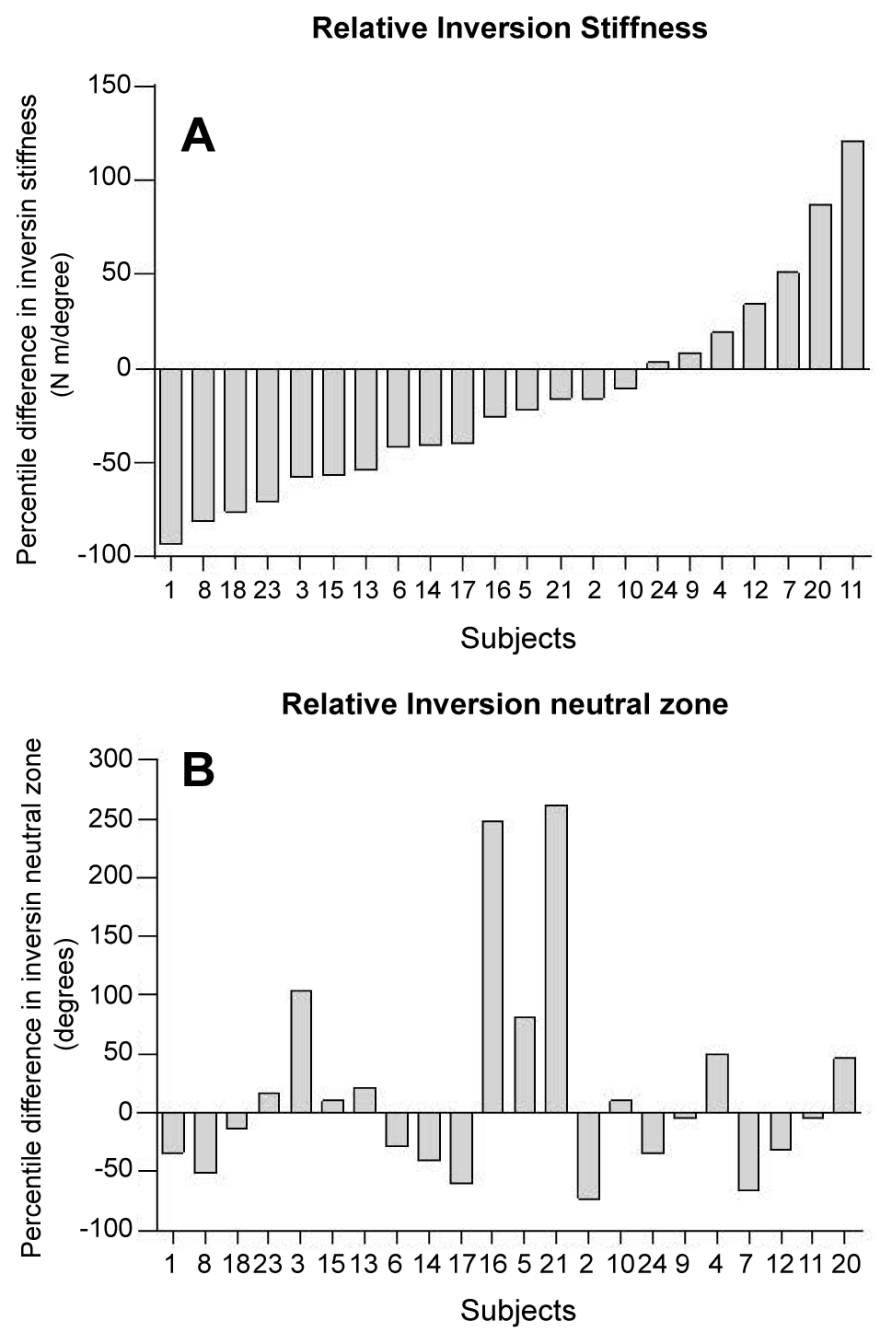
The group data for relative inversion stiffness (A) and inversion neutral zone; (B) of the involved side. The relative values were calculated by subtracting the values of the uninvolved side from the values of the involved side

At baseline, the mean values of the inversion stiffness (0.69 ± 0.37 Nm/degree) on the involved ankle was significantly lower (*p* = 0.011, 95% CI [0.563, 0.544]) than that of uninvolved contralateral sides (0.99 ± 0.41 Nm/degree) (Table 3). With the available sample size, the inversion neutral zone (16.7 ± 7.7 degrees) of the involved ankles was not significantly different (*p* = 0.69) from that of uninvolved contralateral sides (17.5 ± 7.6 degrees), even though the mean value was slightly lower. In addition, no significant difference was observed for eversion stiffness and eversion neutral zone between the involved ankles and uninvolved contralateral side. We further examined the distribution of normalized inversion stiffness and inversion neutral zone in all subjects at baseline. Approximately 77% of individuals in this study presented with decreased inversion stiffness. The inversion stiffness decreased in the majority of the involved ankles compared to the uninvolved ankles within subjects, shown as the negative values in the percentage difference (Figure 4A). However, there were still some involved ankles that showed the opposite. The inversion neutral zone was found increased in only about half of the involved ankles compared to the uninvolved ankles (Figure 4B).

For group comparisons, the normalized values for each dependent variable were calculated and compared. At baseline, the normalized values for each dependent variable between groups were not statistically different (p > 0.05). Following 4-week balance training, the changes in the normalized values for each dependent variable between the baseline and post-intervention was calculated. No significant differences were observed between two groups in changes of the normalized values of the inversion stiffness, eversion stiffness, inversion neutral zone, and eversion neutral zone (Table 4).

## Discussion

The primary finding of this study was that the involved ankle with CAI demonstrated decreased inversion stiffness when compared to the contralateral uninvolved ankle. No difference in the neutral zones between the involved and contralateral uninvolved ankles was found. The 4-week balance training intervention failed to show any significant effect on the passive stiffness and neutral zone measured in this study. This study was unique in that it examined the effect of balance training on the neutral zone in addition to stiffness in the ankles with CAI. The results presented in this paper are a portion of the study results and the results on other measurements will be reported later.

Several investigators have examined ankle joint laxity in patients with CAI, and conflicting results have been reported in the literature [7]. The result of the present study is in agreement with studies that have reported increased inversion laxity in subjects with CAI [8,13,22–25]. Approximately 77% of individuals in our study presented with decreased inversion stiffness which were more than the percentage of subjects with mechanical instability reported in previous studies (from 2.5% to 45%) [13,26–28] (Figure 4A). This observation may be explained in part by differences in subject selection criteria used in the present study. In the past, varied criteria has been used to define CAI and hence different studies may have included non-homogeneous cohorts [29]. We recruited study subjects using a consistent set of inclusion criteria based on their history of recurrent sprains/instability following ankle sprain and responses to CAIT selfreported disability/function questionnaire. The subjects in our study averaged 13.5 points out of 30 points on the CAIT questionnaire indicating a status of severe symptoms. Also, the sample subjects recruited in previous studies were recruited mostly from the active, young university students whereas the average age of the subjects in the present study was 34.3 years that would be comparatively less active than the university students.

The inversion stiffness in the involved ankles of subjects in the present study showed a large variability (Figure 4A), which may be the result of various pathological alterations in the ankle joint after sprain injury. The passive stability of the human ankle joint complex is determined by the congruity of the articular surfaces and ligamentous restraints whereas the dynamic stabilization is provided by the musculotendinous structures [2]. Lateral ankle sprains commonly occur in a forced plantar flexion and inversion position of the ankle during landing on an uneven surface. An ankle sprain may lead to tear, laxity, or weakness of one or more ligamentous restraints, which may lead to decreased static joint stability, recurrent ankle sprains, and limitations in function [30]. Persistent laxity or decreased stiffness after an ankle sprain may be caused by alteration in the fibrous nature and crimp pattern of the ligaments during the healing process [31]. McKay et al. reported that nearly 55% of individuals who sprain their ankle do not seek treatment and that may partially explain why in some individuals the ligaments of the ankle may not heal appropriately [32]. Also, early return to activity or insufficient ligamentous tissue healing can result in improper alignment of the collagen fibers along the principle axis of stress experienced by the ligaments that may lead to increased laxity of the joint [31]. On the contrary, some researchers have proposed that immobilization during the healing process may lead to scar tissue formation that may result in decreased load capacity of the ligament and alterations in sensorimotor system [33,34]. The presence of scar tissue/adhesions in the ligament or joint capsule may decrease the arthro kinematic accessory motions of the joint [35,36] and thus contribute to decreased flexibility or increased stiffness of the joint. Increased peroneal muscle tone, mediated through the gamma motor neuron system has also been hypothesized to explain the stiffness of the ankle joint in some subjects [37]. Extensive future research is required to further examine the influence of the above-mentioned conditions on mechanical characteristics of the joint.

To the best of our knowledge, only one study reported laxity values using a similar device under comparable testing conditions [19]. Direct comparison of the stiffness data measured in our study (average value of the slope of loading portion of the load-displacement curve) with laxity values reported in their study is impossible, as they reported only peak passive resistance torque and maximum inversion range of motion. Our method of measuring the mechanical characteristics, namely inversion-eversion stiffness and neutral zone for the ankle joint in subjects with CAI is similar in principle to those reported in the literature [13,18,20,38,39]. The load-displacement curve of the ankle joints measured in past studies as well as the present study has demonstrated a non-linear pattern. We identified and measured the low-loading range on the load-displacement curve and referred to it as the neutral zone, dividing it further into inversion and eversion neutral zone in respective directions of ankle motion (Figure 3). Our sample population failed to show any difference in the either neutral zones between the involved and contralateral uninvolved ankles. The enlargement and restriction in the inversion and eversion neutral zones was seen in roughly equal number of subjects, thereby failing to show any differences between the involved and contralateral uninvolved ankles (Figure 4B). There were some subjects who did not demonstrate enlarged neutral zones but showed increased joint stiffness. Such phenomenon may be the result of capsular adhesions which leads to restricted neutral zone, and ligamentous laxity which can lead to a decrease in joint stiffness [37]. Therefore, enlarged neutral zone should not be automatically assumed in patients with CAI and based on the results obtained in this study, surgeons should be careful when performing surgery for excessive laxity in patients with CAI. The influence of ligament laxity, capsular adhesions, or adaptations in arthrokinematics on the neutral zone cannot be precisely determined in the present study. Future research needs to further investigate the relationship between ligamentous and capsular contributions toward ankle joint stiffness.

In the present study, the 4-week balance training failed to show any significant effect on the passive stiffness or neutral zone between groups. This result is consistent with the finding of McKeon et al., which was the only past study that examined the effect of balance training on ligamentous laxity and stiffness of the ankle [16]. McKeon and colleagues reported no changes in laxity measures in those who underwent balance training. They suggested that the improvement in coordinative control of the shank and rear foot during gait following balance training were due to the functional changes within the sensorimotor system rather than local changes at the ankle. Traditionally, taping and bracing have been used to improve stability and prevent recurrent ankle injury in patients with CAI. The beneficial effects of ankle taping have been attributed to enhanced proprioception and mechanical restriction, but there is no indication of restoring ligamentous stability [40].

Our study had limitations that should be addressed in future studies. First, the testing of ankle flexibility characteristics in this study was limited to inversion/eversion without anterior drawer testing. It was therefore limited in terms of representing the status of the ATFL in ankles with chronic CAI. Measurements of fibular position, hypo- or hypermobility of the ankle, gender differences, and muscle activation during the testing were not recorded and could have been the limiting factors in this study. Second, the leg and ankle were supported and the subjects were instructed to relax their leg muscles during the testing. Thus based on our testing method, we also cannot determine ligamentous and capsular contributions toward ankle joint stiffness which may be possibly measured with indwelling strain transducers. Third, the balance training program may not have been of sufficient duration to bring changes in the mechanical characteristics of the ankle. In the literature, balance training programs for patients with CAI have been generally prescribed for 3–5 sessions per week for 4–8 weeks. It would be interesting to identify the effect of 6 or 8 weeks balance training program on the mechanical characteristics of the ankle. Lastly, relatively small sample size in this study limited the statistical power of our analyses and could lead to a type 1 statistical error.

## Conclusion

We evaluated the effect of balance training on mechanical characteristics in the ankles with CAI. Our results were in agreement with previously published studies that have reported decreased inversion stiffness in the involved ankle with CAI when compared to the contralateral uninvolved ankle. In addition, no difference in the neutral zones between the involved and contralateral uninvolved ankles was found. The 4-week balance training program failed to show any significant effect on the passive stiffness and neutral zone measured in the relatively small sample size of this study. Further research with additional stiffness measures along with functional tests, larger sample size, and progressive balance training exercises is needed to identify if the mechanical characteristics of chronic unstable ankles can be altered.

## Figures and Tables

**Table 1:** Balance Training Program (A/P – anterior and posterior; M/L – medial and lateral; CW – clockwise; CCW – counter clockwise)

Balancing Component	Activity	Stability level	Number of sets	Duration	Number of repetitions
**Static**	Single-leg stand	6	3	30	-
	Single-leg stand	2	3	30	-
**Dynamic**	A/P tilting	2	3	-	6
	M/L tilting	2	3	-	6
	CW circular movement	2	1	-	10
	CCW circular movement	2	1	-	10

**Table 2:** Subject demographics

	Intervention Group (n = 11)	Control Group (n = 11)
Age, years	33.5 ± 6.6	35.1 ± 9.3
Gender, Male/Female	4/7	3/8
Height, cm	172.7 ± 6.1	168.4 ± 10.7
Mass, kg	77.1 ± 13.2	76.0±14.6
CAIT questionnaire score	12.7 ± 2.3	14.2 ± 4.4
Reports an episode of rehabilitation following ankle sprain, %	54	62
Time since last ankle giving-way, months	4.5±1.9	4.5±2.1

CAIT: Cumberland Ankle Instability Tool. Values are mean ± standard deviation unless otherwise indicated

**Table 3:** Dependent variables at baseline for all subjects in the study

	All subjects (n = 22)		
Variables	Involved Ankle	Uninvolved Ankle	*p*-value
Inversion stiffness (Nm/degree)	0.69 ± 0.37	0.99 ± 0.41	**0.011**
Eversion stiffness (Nm/degree)	0.91 ± 0.54	0.87 ± 0.43	0.727
Inversion neutral zone, (degree)	16.7 ± 7.7	17.5 ± 7.6	0.694
Eversion neutral zone (degree)	12.6 ± 6.6	15.1 ± 4.7	0.153

**Note:** Values are mean ± standard deviation

**Table 4:** Group comparison of the difference in normalized dependent variables at post-intervention

Variables	Intervention Group (n = 11)	Control Group (n = 11)	*p*-value
Change in normalized inversion stiffness, Nm/degree	−0.09 ± 0.16	−0.04 ± 0.16	0.846
Change in normalized eversion stiffness, Nm/degree	0.36 ± 0.17	−0.54 ± 0.17	0.460
Change in normalized inversion neutral angle, degrees	−2.59 ± 3.06	0.38 ± 3.06	0.501
Change in normalized eversion neutral angle, degrees	−3.7 ± 2.4	−1.4 ± 2.4	0.503

**Note:** Values are mean ± std. deviation
